# Falls and Alzheimer Disease

**DOI:** 10.20900/agmr.20240001

**Published:** 2024-02-14

**Authors:** Abigail L. Kehrer-Dunlap, Audrey A. Keleman, Rebecca M. Bollinger, Susan L. Stark

**Affiliations:** Program in Occupational Therapy, Washington University in St. Louis School of Medicine, 4444 Forest Park Ave., Box 8505, St. Louis, MO 63110, USA

**Keywords:** Alzheimer disease, aging, intervention, older adults

## Abstract

Falls are the leading cause of injury, disability, and injury-related mortality in the older adult population. Older adults with Alzheimer disease (AD) are over twice as likely to experience a fall compared to cognitively normal older adults. Intrinsic and extrinsic fall risk factors may influence falls during symptomatic AD; intrinsic factors include changes in cognition and impaired functional mobility, and extrinsic factors include polypharmacy and environmental fall hazards. Despite many known fall risk factors, the high prevalence of falls, and the presence of effective fall prevention interventions for older adults without cognitive impairment, effective fall prevention interventions for older adults with AD to date are limited and inconclusive. Falls may precede AD-related cognitive impairment during the preclinical phase of AD, though a narrow understanding of fall risk factors and fall prevention interventions for older adults with preclinical AD limits clinical treatment of falls among cognitively normal older adults with preclinical AD. This mini review explores fall risk factors in symptomatic AD, evidence for effective fall prevention interventions in symptomatic AD, and preclinical AD as an avenue for future falls research, including recommendations for future research directions to improve our understanding of falls and fall risk during preclinical AD. Early detection and tailored interventions to address these functional changes are needed to reduce the risk of falls for those at risk for developing AD. Concerted efforts should be dedicated to understanding falls to inform precision fall prevention strategies for this population.

## INTRODUCTION

Falls among community-dwelling older adults are a public health crisis. Falls are the leading cause of injury, disability, and injury-related mortality in the older adult population [1]. Approximately 30% of community-dwelling older adults experience one or more falls annually [2,3]. Consequences of falls include fractures, anxiety, and limited activity participation [4,5]. Older adults with neurodegenerative disorders, including Alzheimer disease (AD), Parkinson disease, dementia with Lewy bodies, and Huntington disease, are at a greater risk for falls compared to older adults without neurodegenerative disorders [6]. Given the growing prevalence and significant impact on global health systems, falls among older adults with AD pose a particularly pressing concern [7,8]. Older adults with AD are more than twice as likely (60%–80%) to experience a fall compared to cognitively normal (CN) older adults [9,10]. Older adults with AD who fall experience greater incidence of injurious falls [11,12] and are five times more likely to be institutionalized compared to older adults with AD who do not fall [4,13]. Approximately one in nine people aged 65 and older are currently living with AD, and this prevalence is expected to increase exponentially as the number of older adults worldwide continues to rise [14,15]. Therefore, an improved understanding of falls in AD is a crucial step toward precision fall prevention strategies for this population. A growing body of evidence suggests that an increased risk of falls and functional mobility impairments, such as slowing gait and worsening balance, may precede AD-related cognitive decline during the preclinical phase of AD [16–19]. It is important to understand falls and fall risk factors across the AD continuum, particularly during preclinical AD, as this may enable early preventative treatment of falls prior to the onset of cognitive impairment. Therefore, the objective of this mini review is to provide a concise yet informative update on current evidence related to falls among older adults with AD. The mini review format is ideal to address this topic, as it offers a focused approach to a specific research area and provides an easily accessible summary of relevant findings that may benefit both researchers and healthcare professionals working with older adults across the AD continuum [20]. In this mini review, we will first provide an overview of AD symptoms that can lead to an increased risk of falls, followed by a review of interventions to address falls among older adults with symptomatic AD. Finally, we will examine the preclinical phase of AD as a potential target for future fall prevention research.

## THE ALZHEIMER DISEASE CONTINUUM

AD is a progressive, degenerative brain disorder. Pathological changes of symptomatic AD include abnormal accumulation of proteins, such as beta-amyloid and tau, in the brain that leads to neuronal damage and a decrease in overall brain volume over time [21]. The AD continuum is defined by three phases: preclinical AD, mild cognitive impairment, and dementia due to symptomatic AD [8]. The hallmark symptoms of AD include memory loss, behavioral changes, and safety concerns, including an increased likelihood of falling [8,13,22]. An area that holds promise for understanding the AD-related pathologic and functional changes that precede cognitive decline is the preclinical phase of AD [23]. Individuals with preclinical AD are CN but have brain pathology consistent with symptomatic AD [24]. The preclinical phase of AD can last for years or even decades before progression to symptomatic AD, and it is estimated that 30% of older adults are living with preclinical AD [25]. Advances in neuroimaging and biomarker research have provided valuable insights into the neurophysiological changes that occur during this period of AD [26–29]; in addition to a greater risk of falls [16,17], the presence of AD biomarkers, consistent with preclinical AD, have been associated with functional impairments in complex activities [30], greater dual task interference [31], poorer driving performance [32,33], and sleep disturbances [34]. Therefore, subtle brain changes that begin during the preclinical phase of AD may contribute to an increased risk of falls that persists as AD progresses [16]. Understanding changes that occur in this clinically silent period of AD may improve screening for the progression from preclinical to symptomatic AD.

## INTRINSIC AND EXTRINSIC RISK FACTORS FOR FALLS IN SYMPTOMATIC ALZHEIMER DISEASE

Falls during symptomatic AD are associated with multiple intrinsic and extrinsic risk factors [35]. The risk of falls increases with advancing cognitive impairment [36]. Progressive challenges, including impaired judgment, memory, and executive functioning, may undermine an individual’s capacity to maintain safety awareness or manage complex situations [36]. Delirium, confusion, and disorientation can further compromise the individual’s ability to discern potential fall risks within their physical environment [37,38]. These symptoms also often contribute to wandering behaviors, diminished spatial awareness, and slowed reaction times [38,39]. Dual tasking, or the concurrent execution of a motor-motor or motor-cognitive task [40], is often impaired in older adults with AD and has been linked to an increased risk of falling [41]. This may be attributed to prioritizing the cognitive demands of a secondary task, such as calling a phone number or sustaining a conversation; it may also be due to decreased gait performance, such as decreased cadence and stride length, while attempting to negotiate the task for older adults with AD [42,43]. Additionally, mental health concerns such as depression and anxiety are common among older adults with AD and often contribute to reduced attentional control, fatigue, weakness, agitation, and psychomotor symptoms that impede reaction times, all of which are risk factors for falls [44–46].

The systems and areas of the brain that impact cognition are closely related to those that impact motor function [47,48]. Impairment in cognition, especially higher-level cognition such as executive function, is thought to impact functional mobility (i.e., gait and balance) and muscle strength through impaired motor planning, motor control, attention, and sensory integration [47,48]. AD pathology may influence motor function by disrupting the neural networks that are integral for planning and executing various motor behaviors [49]. Gait parameters, such as decreased stride speed and length, become increasingly impaired as AD progresses [42]. Additionally, declines in physical strength and body composition are associated with AD progression and increase one’s risk of falls [22,49]. Sensory loss is also common in symptomatic AD and contributes to impairments in gait and balance and an increased risk of falls; of note, impaired visual acuity and perception, as well as peripheral sensation, are frequently impaired in AD [50,51]. Dynamic balance (i.e., ability to maintain balance while moving) and dual task conditions require the interaction of cognition and motor functions and are particularly compromised in AD [47,52–54]. In fact, declines in motor and functional mobility have been shown to occur prior to the onset of cognitive impairment in AD and increase older adults’ risk of falls [16,18,50,53,55].

Several extrinsic fall risk factors exist for older adults with AD. They are often prescribed medications for symptoms associated with AD such as depression, anxiety, sleep irregularities, and problematic behaviors, many of which alone have side effects known to increase risk of falls [35,56]. For example, the side effects of psychotropic drugs (antidepressants, antipsychotics, sedatives), acetylcholinesterase inhibitors (cognitive enhancers), and benzodiazepines (sedatives) include weakness, motor disturbances, dizziness, vertigo, depression, and falls [56–58]. In addition, polypharmacy, or taking four or more prescription medications, is a known risk factor for falls due to increasing drug-drug interactions and likelihood of experiencing side effects among community-dwelling older adults [59], including those with AD [60]. Environmental hazards are another type of extrinsic fall risk factor for older adults with AD [38]. Many falls among older adults with AD occur within the home environment [38]. Environmental hazards that may contribute to falls in the home for this population include slippery or uneven flooring, stairs without railings, or furniture of inappropriate heights (e.g., beds, toilets) [38,61]. Furthermore, inadequate lighting or improper footwear may lead to falls among individuals with cognitive impairment, particularly among older adults with AD who have visual impairments or decreased sensation [22,62].

Collectively, the above intrinsic and extrinsic factors can increase the risk of falling for older adults with AD. These factors often interact to increase fall risk even more. In reality, one’s risk of falling is often multifaceted rather than attributable to a single factor [63,64]. For example, an older adult with AD with impaired judgment and lower extremity weakness may experience a fall while descending stairs with no handrail. To address multifaceted fall risks, interventions that are robust to target multiple domains of risk factors are necessary.

## FALL PREVENTION INTERVENTIONS FOR OLDER ADULTS WITH ALZHEIMER DISEASE

Despite many known fall risk factors, the high prevalence of falls, and the presence of effective fall prevention interventions for older adults without cognitive impairment, effective fall prevention interventions for older adults with AD to date are limited and inconclusive [10,53,65–68]. Some studies suggest that the critical components of adherence to fall prevention interventions, such as structured home-based exercise programs and home hazard removal programs, for older adults with AD include support from clinicians, particularly occupational and physical therapists, and caregivers [69–72]. Exercise interventions often target major fall risk factors of poor balance and muscle weakness. Exercise interventions are feasible and improve balance among older adults with AD [72,73], but there is limited evidence on their effectiveness in reducing falls in this population [66,74–76]. In addition, interventions to reduce home hazards or fall risks in the environment (i.e., tripping hazards, cluttered pathways, slippery floors) are effective among older adults at risk of falling [77], especially when they are delivered by an occupational therapist with community-dwelling older adults [78]. However, the effectiveness of home hazard removal and home modifications is less well-established among older adults with AD. Insufficient evidence indicates that home modifications alone are sufficient to reduce the incidence of falls among older adults with AD, though they may have greater effectiveness in reducing falls when combined with other approaches, such as exercise [68,79], but not with Vitamin D supplementation, for cognitively impaired older adults [76]. Home-based systems, including bed-exit alarm systems and automatic path lighting, have demonstrated effectiveness in reducing the risk and incidence of falls [79,80]. Additionally, efforts to limit wandering, such as monitoring devices, concealed doorways, and wander gardens, may hold promise for effectively reducing falls among older adults with AD, though these findings have been limited to individuals in residential or institutional settings [79]. While medication review and reducing the number of interacting prescription medications can be effective in reducing falls among CN older adults [59], further investigation into polypharmacy and falls is warranted among older adults with AD pathology to determine best practices for prescribing fall-risk-increasing medications within this population [81].

Interventions that target multiple fall risk factors have been more commonly tested in a clinical trial setting with mixed results [61,67,76,82]. For example, an intervention that included exercise and tailored home hazard removal among community-dwelling older adults with cognitive impairment [61] and an intervention that included medication review, vision correction, exercise, and home hazard modification in people with cognitive impairment did not reduce falls [82]. However, a recent review of fall prevention interventions found that those targeting strength, balance, and cognition (executive function) can improve postural stability and may reduce the risk of falling among older adults with AD [67]; however, additional research is needed to examine the effectiveness of multicomponent fall prevention interventions for older adults with AD.

The evidence on effective fall prevention interventions among community-dwelling older adults with symptomatic AD is limited. The exclusion of older adults with cognitive impairment in clinical trials greatly limits knowledge in this area, and, among studies that include those with symptomatic AD, clinical implications are tempered due to weak study designs [10,53,65–68]. Common challenges specific to symptomatic AD are difficulty controlling for and assessing stage of AD, confounding factors, and the accelerated declines associated with AD, as well as the general aging process and comorbidities. Testing fall prevention interventions in an earlier stage of AD prior to onset of hallmark memory symptoms, such as preclinical AD, may be a particularly promising direction to better understand the effectiveness of fall prevention interventions during the progression of AD.

## PRECLINICAL ALZHEIMER DISEASE: AN AVENUE FOR FUTURE FALLS RESEARCH?

The preclinical phase of AD has gained much attention in recent years as a potential target for intervention for slowing or preventing progression to symptomatic AD [26,83]. Older adults with preclinical AD show no symptoms of cognitive impairment through traditional screening measures but have evidence of AD pathology [26]. Core biomarker abnormalities, including abnormal accumulation of beta-amyloid and tau proteins, may present decades before symptomatic AD [84]. AD biomarkers are typically evaluated through positron emission tomography, magnetic resonance imaging, cerebrospinal fluid, or, in recent developments, blood plasma samples [28,29]. These biomarkers can be used to stage progression from preclinical to symptomatic AD [85]; however, screening for AD biomarkers is costly and time consuming and, thus, is not part of routine clinical care for older adults [86]. As a result, additional work is needed to improve screening and detection of preclinical AD in order for this phase to be a robust target for fall prevention interventions for people with AD.

**Figure 1.**
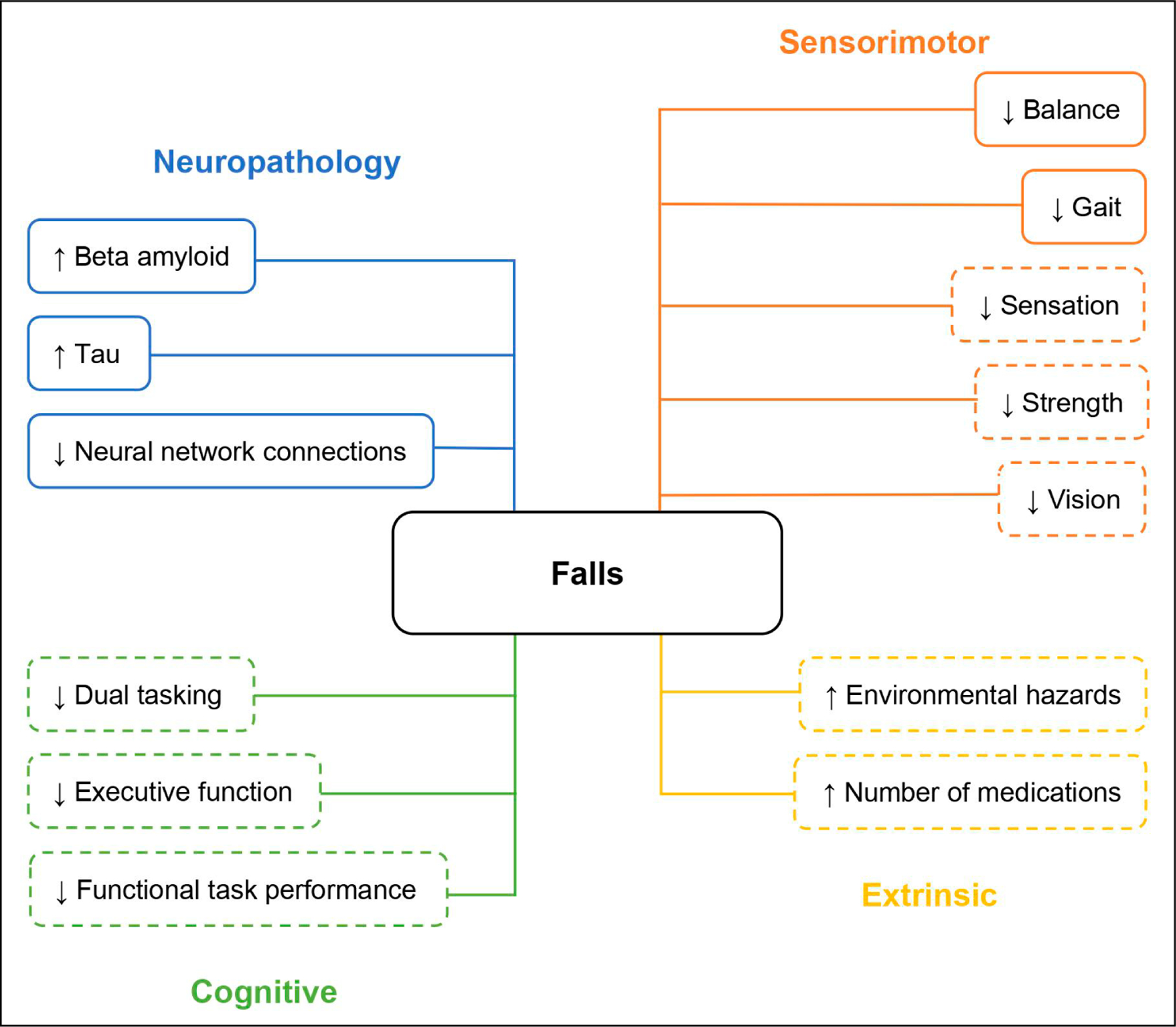
Fall risk factors in the preclinical phase of Alzheimer disease (AD). Note: Solid lines represent well-established risk factors for falls in preclinical AD. Dashed lines represent factors requiring further investigation to determine their roles in influencing falls risk in preclinical AD.

Examining falls may be a simple and inexpensive way to screen for preclinical AD. Emerging research examining falls during preclinical AD has identified potential relationships between increased falls and AD pathology. The presence of AD biomarkers has been associated with greater numbers of falls and decreased time to experiencing a fall compared to older adults without preclinical AD biomarkers [16,17]. Additionally, evidence suggests that worsening balance and slowing gait precede cognitive decline by several years and are linked to AD pathology [18,19,87]. Therefore, balance and gait speed assessments should be integrated into routine clinical evaluations of older adults to identify those who may be at an increased risk of cognitive decline and AD [87]. Dual-task gait may better predict future cognitive decline for older adults with preclinical AD than gait speed alone, although further research is needed to determine whether differences in dual tasking arise during the preclinical phase of AD or exclusively in symptomatic AD [88]. Other sensorimotor fall risk factors, including balance, sensation, strength, and vision, have not been investigated specifically in relation to preclinical AD; therefore, it is difficult to determine whether these risk factors precede or arise following AD-related cognitive impairment. Figure 1 presents a conceptual diagram highlighting fall risk factors that are present in symptomatic AD. It is unknown whether an increased risk of falling during preclinical AD is the result of biological abnormalities, behavioral changes, or a combination of these factors. Gaining a deeper understanding of the mechanisms underlying falls during preclinical AD holds great promise for early detection and intervention strategies.

While addressing the preclinical phase of AD holds potential for falls research, several evidence gaps remain. To our knowledge, no studies have reported fall risk factors specific to the preclinical phase of AD. Knowledge of the intrinsic and extrinsic factors that increase one’s risk of falling may improve targeted screening for preclinical AD. Additionally, the ways in which the circumstances of falls, such as where, when, or how they occur, differ between older adults with and without preclinical AD remain unknown. It is also unclear where falls begin during the progression from preclinical to symptomatic AD, and this knowledge could inform targeted fall prevention interventions.

Understanding falls and changes in functional mobility that begin during preclinical AD may improve detection and inform precision fall prevention interventions for older adults with preclinical AD. Future efforts to understand falls during the preclinical phase of AD should help identify fall risk factors during this phase, which will allow for comparisons of fall risk factors during preclinical and symptomatic AD. In addition, future studies should examine dual-task gait as an improved predictor of future cognitive decline compared to traditional gait speed assessments. While polypharmacy remains a fall risk factor for older adults with and without AD, future research should attempt to elucidate relationships among polypharmacy, falls, and preclinical AD, as the pathologic changes during preclinical AD may interact with prescription medications and increase one’s risk of falls. Longitudinal studies incorporating comprehensive assessments of both cognitive and motor function are needed to better understand the relationship between AD and falls during the preclinical phase. These studies should report circumstantial information about falls, including where, when, and how falls occur, as this information is important to design tailored approaches to fall prevention. These studies should also focus on identifying when falls occur in the progression from preclinical to symptomatic AD. Finally, interventions that prevent or reduce falls, including exercise programs, home fall hazard removal, and multicomponent approaches, warrant further investigation to determine their efficacy among older adults with preclinical AD.

## CONCLUSIONS

Understanding the complex relationship between falls and AD is of utmost importance, as the older adult population is expected to rise exponentially over the next 30 years [15]. Growing evidence suggests that subtle cognitive changes, functional impairments, and falls may precede cognitive decline during the preclinical phase of AD [16,17,30,33]. Early detection of falls and tailored interventions to address functional changes are needed to reduce fall risk for those at risk for developing AD. Future studies should examine fall risk factors, circumstances of falls, and efficacy of fall prevention interventions, such as exercise and home hazard removal, among CN older adults with preclinical AD. Falls among older adults with AD represent a significant public health concern; therefore, concerted efforts should be dedicated to understanding falls to inform precision fall prevention strategies for this population.

## Data Availability

Data sharing is not applicable to this article, as no datasets were generated or analyzed during the current study.

## ABBREVIATIONS

AD: Alzheimer disease
CN: cognitively normal
